# Perceived Family Support, Depression, and Suicidal Ideation among People Living with HIV/AIDS: A Cross-Sectional Study in the Kathmandu Valley, Nepal

**DOI:** 10.1371/journal.pone.0090959

**Published:** 2014-03-06

**Authors:** Rachel M. Amiya, Krishna C. Poudel, Kalpana Poudel-Tandukar, Basu D. Pandey, Masamine Jimba

**Affiliations:** 1 Department of Community and Global Health, Graduate School of Medicine, University of Tokyo, Tokyo, Japan; 2 Department of Public Health, School of Public Health and Health Sciences, University of Massachusetts-Amherst, Amherst, Massachusetts, United States of America; 3 Waseda Institute for Advanced Study, Waseda University, Tokyo, Japan; 4 Everest International Clinic and Research Center, Kathmandu, Nepal; Old Dominion University, United States of America

## Abstract

**Background:**

Depression and suicidal thinking occur frequently alongside HIV/AIDS, triggering profound detrimental impacts on quality of life, treatment adherence, disease progression, and mortality. Yet the psychosocial factors contributing to these psychiatric comorbidities remain underexplored, particularly in the developing country context. This study thus examined different dimensions of perceived family support in relation to depression and suicidal ideation among people living with HIV/AIDS (PLWHA) in Nepal.

**Methods:**

A cross-sectional survey of 322 adult PLWHA residing in the Kathmandu Valley, Nepal was conducted. Data were analyzed using multiple logistic regressions for correlates of Beck Depression Inventory (BDI)-Ia-defined depressive symptoms and suicidal ideation in the past 2 weeks. Perceived family support, measured using the 10-item Nepali Family Support and Difficulty Scale, was entered into separate models, in turn, as a composite score, for each sub-scale (emotional, instrumental, and negative support), and for each individual item.

**Results:**

Overall, 25.5% of participants registered BDI-Ia-defined depression, with significantly lower rates among those with perceived family support scores in the highest (AOR = 0.19; 95% CI = 0.07, 0.55) and middle (AOR = 0.38; 95% CI = 0.17, 0.86) tertiles relative to those with lowest-tertile scores. Meanwhile, 14.0% reported suicidal thinking, with significantly lower rates among those in the highest perceived family support tertile relative to the lowest (AOR = 0.25; 95% CI = 0.07, 0.91). Broken down by support sub-scale, only negative support (i.e. family difficulty) was significant in its correlations with both outcomes – a trend similarly reflected in the item-wise analyses.

**Conclusions:**

Our findings highlight an important role for family support in determining experiences of depression and suicidality among PLWHA. Incorporating family counseling and support services – with special focus on ameliorating negative interaction and bolstering emotional support – into HIV care and treatment services may help to improve mental health along with overall wellness and treatment outcomes for HIV-positive populations in Nepal and similar settings.

## Introduction

With the prolonged life expectancy made possible through introduction of highly active antiretroviral therapy (HAART), mental health issues have come to the fore as a critical problem in people living with HIV/AIDS (PLWHA). Especially common among the several psychiatric conditions comorbid with HIV/AIDS are depression and suicidal ideation [1]–[3]. Though reported rates of depression in PLWHA vary widely depending on regional context, diagnostic criteria, methods of measurement, and study sample, estimates are consistently high across countries – between 12% and 71% as measured by the Beck Depression Inventory (BDI) alone [1]. Against an estimated lifetime depressive disorder prevalence of 6.7% in the general population [4], the burden is at least two to three times higher for PLHWA [1], [2]. Similarly, suicidal ideation, attempts, and completions remain alarmingly common among PLWHA, despite a recorded decline in suicide rates since the advent of HAART in the 1990s to levels comparable with those of other chronic disease-afflicted populations [3].

Worldwide, mental health problems are a serious and growing public health problem, constituting the brunt of the non-fatal burden of disease in 2010. Under this umbrella, depressive disorders represent the leading cause of disability-adjusted life years (DALYs) attributable to mental, neurological, and substance use disorders in high-income and low- and middle-income countries (LMICs) alike [5], and suicide accounts for roughly one million deaths annually worldwide [6]. Yet in the context of HIV, the ramifications are uniquely deleterious. Depressive symptoms in PLWHA are associated with poor adherence to medical appointments and antiretroviral therapy (ART) [7], along with adverse medical outcomes including higher risk for comorbid disorders, faster progression from HIV to AIDS, and higher mortality from both AIDS-related and non-AIDS-related causes [2], [8]. At the same time, depression has been associated with increased HIV risk behaviors [2], [9]. Failure to recognize and address depression in the context of HIV may thus endanger not only the individual but the community as well. On top of this, high rates of suicidal ideation among PLWHA are a serious concern in that suicidal thoughts precede suicidal behaviors. Such psychosocial health problems are critically important to address among PLWHA, particularly inasmuch as they can act syndemically to the detriment of efforts to curb HIV.

Against the heavy burden of psychiatric comorbidities associated with a chronic illness such as HIV/AIDS, social and family support may have a moderating effect and present a promising mechanism through which the mental health needs of PLWHA might fruitfully be addressed. Social resources are generally protective against adverse psychological responses to stressful situations [10]. Indeed, even the mere *perceived* availability of social support can have a direct beneficial impact on health and mood [10], [11] and buffer against suicide risk conferred by chronic disease and other stressful life events [12]. Likewise, the importance of social support in the psychological adjustment to living with HIV/AIDS has also been documented, manifesting in a strong inverse relationship between supportive social interactions and depression in diverse samples of PLWHA [13], [14].

Among the different types of social support, family support is generally one of the most important factors affecting how patients adapt to illness [15]. The perception of a supportive family environment may protect individuals from the potential psychopathological effects stemming from the physical impact of their disease. Moreover, family is frequently the main source of support in times of illness, whether through tangible instrumental support, such as preparing meals and administering medication, or through emotional support. In cancer and end-stage renal disease patients, higher levels of family support are associated with lower levels of depression [16], [17], while lack of family support has been linked with increased suicide rates in chronic disease patients [18]. Similarly, one study among HIV-positive women in the U.S. found that those reporting suicidal thoughts also reported less family cohesion, while higher levels of family cohesion buffered the suicidal thought-inducing potential of HIV-related symptoms [19].

While the beneficial impacts of support from social and family networks are widely recognized, the same intimate relationships can also be a source of tension and discord. Such *negative* social interactions constitute a distinct dimension of social support, with separate and potentially deleterious impacts on mental health [20]–[22]. Importantly, sustained interpersonal strains tend to be more characteristic of relationships with family than with non-related peers, as friendships high in unsupportive elements are generally less likely to be maintained relative to the more obligatory bonds of family [23]. Yet few studies to date have considered the way that each discrete aspect of perceived family support, both negative and positive, may differentially impact mental health. Moreover, while research has highlighted the important mental health impacts of psychosocial variables such as family support in other acute and chronic diseases, few studies have examined these factors in the context of HIV/AIDS [13], [24], [25]. This is particularly true in LMICs like Nepal, where mental health remains a lower priority in general [26]. Similarly, almost all studies of suicidal ideation, attempts, and completions have taken place in the developed as opposed to the developing world, where 85% of suicides occur and where the brunt of the HIV burden lies [3].

Such a myopic perspective may be problematic when extrapolating to other populations, as fundamental differences in the historical, cultural, and sociological fabric of a country can come into heavy play and limit generalizability of findings. In Asian countries, for example, different risk factors are known to play a greater role in suicide, including impulsiveness, financial stress, and interpersonal conflict [3]. In the same vein, the restricted focus of much of the social support research within the United States and other Western countries demands expanded attention in that differences in support-seeking and perceptions of support are closely interwoven into culture [27]. In Asian societies, for example, familial relationships are typically marked by a higher level of responsibility and obligation; consequently, individuals may be more reticent to discuss problems openly out of concern for the potential negative ramifications to the group [28]. Given the high cultural value assigned to family in more collectivistic, interdependent societies and the greater extent to which kin are bound to one another to fulfill emotional and instrumental needs [29], perceived support from family may well be equally or more important to well-being in such settings.

The aim of the present study was thus to examine factors associated with depression and suicidal ideation among PLWHA in the low-income South Asian country of Nepal, with a particular emphasis on elements of perceived family support. In particular, greater understanding of the manifestations and meanings of family support in this context could guide caregivers and policy makers in designing effective psychosocial interventions targeting PLWHA and their family members, with significant implications for the success of HIV prevention and treatment efforts. Such angles may be particularly important in a context of severely constrained resources for mental health.

## Methods

### Study Design and Setting

This cross-sectional study surveyed a community-based sample [30]–[32] of 322 HIV-positive residents of the Kathmandu Valley in Nepal, among the poorest countries in South Asia [33]. From its first reported case of HIV in 1988, Nepal has faced an escalating concentrated epidemic, with key at-risk populations – men who have sex with men, people who inject drugs, female sex workers, and male labor migrants – constituting 58% of all adult HIV infections [34]. As of 2011, national estimates indicate that approximately 43,000 adults (15–49 years) are infected, yielding an overall adult prevalence of about 0.3% in the general population [34]. At the end of 2006, almost 16% of the country’s PLWHA population was residing in the Kathmandu Valley [35], a region in central Nepal comprising three densely populated districts (Kathmandu, Lalitpur, and Bhaktapur) with an estimated population of around 2.5 million in 2011 [36].

### Data Collection

A total of 322 participants were recruited through purposive, convenience sampling techniques, as a detailed database of HIV-positive individuals was not available from which to select randomly. Referrals were obtained through staff members of five local non-governmental organizations (NGOs) working within HIV-positive communities in the study area. Individuals recruited for participation fulfilled the following inclusion criteria: (1) aged 18 to 60 years, (2) self-reported diagnosis of HIV-positive status, and (3) willing provision of written informed consent for voluntary participation.

Data were collected during February and March 2010 as part of the baseline phase of an ongoing Healthy Living Intervention Study headed by the second and third authors. Trained interviewers administered the structured, pre-tested Nepali language questionnaire. Interviews were face-to-face and conducted in a private setting, with each lasting approximately 45–60 minutes.

### Ethics Statement

All participants were briefed on the study procedures with the aid of a prepared information sheet, after which each was asked to provide written informed consent prior to being interviewed. Numerical codes were used in place of names in all records to ensure confidentiality. Regarding sample selection procedures, the minimum age of 18 was used as the formal inclusion criterion because this is the legal age of majority in Nepal. Ultimately, however, the youngest participant was 20 years old. Both the Research Ethics Committee of the Graduate School of Medicine at the University of Tokyo and the Nepal Health Research Council reviewed and approved all study protocols and procedures.

### Measures

*Perceived family support*. Perceived family support is defined herein as the felt provision of different forms of emotional and instrumental services and assistance from family members, along with negative, or *un*supportive, forms of family interaction, within the past year, as measured using the 10-item Nepali Family Support and Difficulty Scale (Cronbach’s alpha = 0.87). For each item, participants were asked to rate how true each statement was for their own family on a four-point Likert scale ranging from “Not at all” (0) to “All the time” (3). The scale was developed specifically for use in Nepal. After reversing the scores for negatively formulated items (i.e., measuring negative family interaction), the total score was derived by summing all items, with higher scores indicating greater perceived family support (instrument range: 0–30). For the analysis, total scores were categorized into *low* (0–22), *moderate* (23–26), and *high* (27–30) levels of perceived support by tertiles.

Total sub-scores for each of the different types of family support measured three dimensions of social exchange: *emotional support* (4 items; Cronbach’s alpha = 0.79; e.g., “How much does your family show love and caring for you?”), *instrumental support* (2 items; Cronbach’s alpha = 0.66; e.g., “How much support do you get when you are sick?”), and *negative interaction* (4 items; Cronbach’s alpha = 0.74; e.g., “How much do you feel disliked by your family?”). For the analysis, total sub-scores within each support category were again classified as *low*, *medium*, or *high* by tertiles.

#### Depression and suicidal ideation

The 21-item BDI-Ia, Nepali version [37], [38], was used to assess depression in participants over the prior 2 weeks (Cronbach’s alpha = 0.89). Items are scored on a four-point Likert scale with an instrument range of 0 to 62. The scale has been validated for use in Nepal [37] with clinical DSM-IV diagnoses of major depressive disorder (area under the curve [AUC] 0.92), based on which a score of 20 or higher suggests moderate to severe depressive symptoms with the need for mental health intervention (sensitivity = 0.73, specificity = 0.91) [37]. This cut-off score is intended only to reflect symptom burden at the level requiring intervention and does not indicate diagnosis of major depressive disorder.

Assessment of suicidal ideation was based on BDI-Ia item #9 (“During the past 2 weeks, have you thought about ending your life?”), which was used as a dichotomous variable indicating the presence or absence of suicidal thoughts or wishes. Suicidal ideation endorsement was defined as responding to BDI-Ia item #9 with either (1) “I have thoughts of killing myself but I would not carry them out,” (2) “I would like to kill myself,” or (3) “I would kill myself if I had the chance”. Also included on the questionnaire were items asking about ever-experience of suicidal ideation and number of suicide attempts (if any) since being diagnosed with HIV.

#### Sociodemographic, clinical, and psychosocial characteristics

Measures of key sociodemographic, HIV-specific clinical, and psychosocial variables were included in the analyses as control variables.

Standard single questionnaire items assessed basic sociodemographic, psychosocial, and HIV-specific clinical variables. Body mass index (BMI) was calculated from the measurement of height and body weight, with underweight defined as a BMI of less than 18.5 kg/m^2^ according to the WHO BMI classification standard for Asians [39].

A modified 17-item version of the HIV Symptom Index (HSI) [40] was used to measure HIV symptom burden based on a 1-month recall period (Cronbach’s alpha = 0.90). Though the original version of this scale assesses the presence and degree of 20 symptoms commonly experienced by PLWHA, three non-somatic symptoms (*Felt sad, down or depressed*; *Felt nervous or anxious*; and *Difficulty falling or staying asleep*) were omitted for the purposes of this study to focus on the somatic aspect and avoid overlap with the measure of depressive symptoms. Participants reported whether each symptom was present, and if so, whether it was bothersome, by using a five-point Likert scale ranging from “I do not have this symptom” (0) to “I have it and it bothers me a lot” (4). Each item was dichotomized into absent/not bothersome (0–2) vs. present and bothersome (3–4) and the resulting scores summed to obtain a bothersome symptom count, with a possible range of 0–17 [40]. For the analysis, total counts were dichotomized into lower and higher symptom burdens by the median (2).

A modified 7-item version of the AIDS-related Stigma Scale [41] was used to assess internalized AIDS stigma (Cronbach’s alpha = 0.75). Responses were given dichotomously (0 = disagree, 1 = agree); scale scores represent the sum total of endorsed items, with higher scores indicating more negative attitudes or perceived discrimination (instrument range: 0–7). For the analysis, total scores were dichotomized into lower and higher levels of stigma by the median (4).

### Statistical Analysis

Descriptive statistics were calculated for demographic and other relevant characteristics of the study sample. Multiple logistic regression analysis was then used to examine factors associated with depressive symptoms and suicidal ideation, with particular emphasis on the association between level of perceived family support and the two mental health outcomes. Separate multivariate models were also separately constructed to assess potential effects of each of the family support sub-scores and individual scale items on depression and suicidal ideation among the participants, adjusting for the same set of potential confounders. Additional logistic regression analysis was performed to explore factors associated with family support, with total family support collapsed into a dichotomous categorical variable based on the overall median score (25).

Independent variables were entered into each regression using a direct (simultaneous) entry method. All major sociodemographic characteristics and other factors having previously established or theoretically feasible associations with the dependent variables were included as covariates or potential confounders in the analyses. Additionally, interactions between perceived family support and each of the other variables included as covariates were tested by evaluating the statistical significance of the corresponding first-order cross product terms in separate corresponding models; as none of the interaction terms was statistically significant, they were excluded from the final models. Variance inflation factors (VIFs) were low (<2.0) in all cases, indicating no problematic multicollinearity among independent variables. All statistical tests were 2-sided, evaluated as significant at the p<0.05 level, and executed using SPSS version 18.0 for Macintosh (SPSS Inc., Chicago, IL, USA).

## Results

### Background Characteristics

Table 1 presents the basic background characteristics of the surveyed group of 322 PLWHA. Participants were 58% male and had a median age of 33 (interquartile range [IQR] = 30, 39) years; 81% had at least some formal education, with 60% educated at an above-primary level, and 75% were employed in some capacity. Median period since testing HIV-positive was 53 (IQR = 25, 85) months and 73% of participants were on ART at the time of survey, among whom the median length of time on treatment was 24 (IQR = 13, 33). Based on BMI (<18.5), 9.2% of participants were underweight. Median bothersome HIV symptom count was 2 (IQR = 0, 6), and median internalized AIDS-related stigma scale score was 4 (IQR = 2, 6). Overall, 41% of participants had a lifetime history of injecting drug use, while 85% had used some form of illicit drug in the past six months. Only 18% had not disclosed their HIV status to any of their family members. Median perceived family support, meanwhile, was 25 (IQR = 19,27), out of a total possible score of 30.

**Table 1:** Background characteristics of participants (N = 322).

Characteristic	n	%
***Sociodemographics***		
**Gender,** male	185	57.5
**Age** a, *median (IQR)* years	33 (30, 39)	
**Current marital status**, Married	221	68.6
**Having any children**, Yes	220	68.3
**Education level** b ^,^ c		
No formal education	58	18.6
Primary (1–5 yrs.)	68	21.8
Lower secondary (6–10 yrs.)	157	50.3
Higher secondary and above (11+ yrs.)	29	9.3
**Employment status** d, Employed	240	75.0
***Clinical and psychosocial characteristics***		
**Time since first testing HIV+** e, *median (IQR)* months	53 (25, 85)	
**Currently on ART**, Yes	234	72.9
Time on ART^f^, *median (IQR) months* g	24 (9, 39)	
**Underweight (BMI<18.5)** h, Yes	29	9.2
**Any illicit drug use (last 6 months)**, Yes	47	14.6
**Bothersome HIV symptom count**, median (IQR)	2 (0, 6)	
**Internalized AIDS stigma score**, median (IQR)	4 (2, 6)	
**Disclosure of HIV status to any family members** i, Yes	260	82.0
***Family support and mental health variables***		
**Perceived family support**		
Low (Total score: 0–22)	117	36.3
Moderate (Total score: 23–26)	113	35.1
High (Total score: 27–30)	92	28.6
**Depressive symptoms**, Moderate to severe (BDI-Ia>20)^j^	82	25.5
**Suicidal ideation (last 2 weeks)**, Yes	45	14.0
**Ever thought about ending life since testing HIV+**, Yes	138	42.9
**Ever attempted suicide since testing HIV+**, Yes	54	16.8

IQR, interquartile range; *ART,* antiretroviral therapy; BMI, body mass index; BDI, Beck Depression Inventory.

a Two individuals did not respond to this item.

b Three individuals did not provide any information about their education level; seven individuals who reported some formal education did not indicate their years of schooling.

c Education level categories were defined based on the structure of the Nepalese education system.

d Two individuals did not respond to this item.

e Five individuals did not respond to this item.

f One individual did not respond to this item.

g Median (IQR) was calculated for the 234 individuals on ART at the time of survey.

h Six individuals did not respond to this item.

i Five individuals did not respond to this item.

j A score of 20 or more on the Beck Depression Inventory indicates moderate-to-severe depression with the need for mental health intervention, based on clinical validation of the scale in Nepal (sensitivity = 0.73, specificity = 0.91) [37].

### Depression and Suicidality Prevalence Rates

Among all participants, 26% met the BDI-Ia threshold for depression. Suicidal ideation in the previous 2 weeks was reported by 14% of respondents. Taking a broader perspective, 43% had *ever* thought about ending their lives and 17% had actually attempted suicide since being diagnosed with HIV, with 35 individuals reporting more than one such suicide attempt (Table 1).

### Factors Associated with Depression and Suicidal Ideation

Identified correlates of depression (BDI-Ia≥20) and suicidal ideation (BDI-Ia item #9>0) from multivariate regression models are presented in Table 2. High perceived family support relative to low perceived family support was inversely associated with both depression (adjusted odds ratio [AOR] = 0.19; 95% confidence interval [CI] = 0.07, 0.55) and suicidal ideation (AOR = 0.25; 95% CI = 0.07, 0.91) risks. In the case of depressive symptoms, lower rates were linked to even moderate levels of perceived family support relative to low levels (AOR = 0.38; 95% CI = 0.17, 0.86). Against the observed inverse relationship with perceived family support, four further variables were positively associated with both psychiatric comorbidities: higher bothersome HIV symptom count (depression: AOR = 4.28; 95% CI = 2.09, 8.80/suicidal ideation: AOR = 3.71; 95% CI = 1.54, 8.94); being on ART for 2 years or less compared to not being on ART (depression: AOR = 2.92; 95% CI = 1.13, 7.51/suicidal ideation: AOR = 3.61; 95% CI = 1.15, 11.39); reporting any illicit drug use in the past 6 months (depression: AOR = 2.86; 95% CI = 1.07, 7.66/suicidal ideation: AOR = 3.63; 95% CI = 1.23, 10.73); and being unemployed (depression: AOR = 2.17; 95% CI = 1.01, 4.66/suicidal ideation: AOR = 3.70; 95% CI = 1.58, 8.68).

**Table 2:** Multiple logistic regression analysis^a^ of factors associated with depression and suicidal ideation among participants (N = 292^b^).

	Depression (BDI-Ia≥20)		Suicidal ideation (BDI-Ia item #9>0)	
Variable	AOR	(95% CI)	AOR	(95% CI)
**Gender**				
Female	0.61	(0.25, 1.48)	0.40	(0.14, 1.13)
Male (Ref)	1.00		1.00	
**Age (years)**				
34–60	2.01	(0.95, 4.25)	0.62	(0.26, 1.51)
0–33 (Ref)	1.00		1.00	
**Current marital status**				
Married	1.43	(0.64, 3.17)	0.82	(0.33, 2.07)
Unmarried (Ref)	1.00		1.00	
**Any children**				
Yes	1.24	(0.55, 2.78)	0.85	(0.33, 2.22)
No (Ref)	1.00		1.00	
**Education level**				
Secondary or higher	0.68	(0.33, 1.40)	0.54	(0.23, 1.27)
Primary or lower (Ref)	1.00		1.00	
**Employment status**				
Employed	0.46	(0.22, 0.99)*	0.27	(0.12, 0.63)**
Unemployed (Ref)	1.00		1.00	
**Time since first testing HIV+, months**				
54–258	1.07	(0.53, 2.19)	0.88	(0.38, 2.08)
0–53 (Ref)	1.00		1.00	
**Months on ART**				
25–120	1.85	(0.63, 5.46)	2.92	(0.77, 11.11)
0–24	2.92	(1.13, 7.51)*	3.61	(1.15, 11.39)*
Not currently on ART (Ref)	1.00		1.00	
**Underweight (BMI<18.5)**				
Yes	3.29	(1.06, 10.20)*	1.21	(0.34, 4.30)
No (Ref)	1.00		1.00	
**Any drug use, last 6 mos.**				
Yes	2.86	(1.07, 7.66)*	3.63	(1.23, 10.73)*
No (Ref)	1.00		1.00	
**Bothersome HIV symptom count**				
3–16	4.28	(2.09, 8.80)**	3.71	(1.54, 8.94)**
0–2 (Ref)	1.00		1.00	
**Internalized AIDS stigma score**				
5–7	3.37	(1.66, 6.87)**	1.81	(0.77, 4.24)
0–4 (Ref)	1.00		1.00	
**Disclosure of HIV status to any family members**				
Yes	2.05	(0.80, 5.23)	1.56	(0.52, 4.64)
No (Ref)	1.00		1.00	
**Perceived family support**				
High (Total score: 27–30)	0.19	(0.07, 0.55)**	0.25	(0.07, 0.91)*
Moderate (Total score: 23–26)	0.38	(0.17, 0.86)*	0.46	(0.17, 1.19)
Low (Total score: 0–22) (Ref)	1.00		1.00	

*AOR,* adjusted odds ratio. *CI,* confidence interval. *ART,* antiretroviral therapy; BDI, Beck Depression Inventory; BMI, body mass index.

a In addition to perceived family support as the primary independent variable of interest, all measured sociodemographic characteristics and other factors having previously established or theoretically feasible associations with the dependent variables were included as covariates or potential confounders in the analyses, as listed within the table.

b After accounting for the collective set of missing values associated with the variables included in the multivariable model, data from 292 participants were analyzed.

*p<0.05;

**p<0.01.

Higher rates of depression were also associated with higher levels of internalized AIDS stigma (AOR = 2.56; 95% CI = 1.33, 4.93) and being underweight (AOR = 3.29; 95% CI = 1.06, 10.20). Older-age participants were similarly inclined toward greater rates of depressive symptoms, but the association did not reach statistical significance.

### Family Support as a Correlate of Depression and Suicidal Ideation

Table 3 presents the results for multiple logistic regression analysis of sub-scale scores and individual items from the perceived family support scale associated with depression and suicidal ideation among participants. Of the three different sub-types of support measured, only negative interaction was significantly associated with either psychological disturbance measure; those reporting high levels of negative interaction with their family were over three-and-a-half times more likely to be depressed (AOR = 3.70; 95% CI = 1.68, 8.18) and over five-and-a-half times more likely to report suicidal ideation (AOR = 5.58; 95% CI = 2.08, 14.96) than were their counterparts with low levels of negative family interaction. Those reporting high levels of emotional support were also over twice as likely to register depression than were those reporting low levels of such support, though this association did not reach statistical significance.

**Table 3:** Multiple logistic regression analysis^a^ of individual perceived family support items associated with depression and suicidal ideation among participants (N = 292^b^).

Item^c^	Depression(BDI-Ia≥20)		Suicidal ideation(BDI-Ia item #9>0)	
	AOR	(95% CI)	AOR	(95% CI)
***Emotional support***				
Feeling shown love and caring by family	0.68	(0.46, 0.99)*	0.64	(0.41, 0.99)*
Feeling have an important role in family	0.94	(0.64, 1.38)	0.70	(0.46, 1.09)
Feeling involved in family decision making	0.88	(0.64, 1.22)	0.69	(0.47, 1.02)
Feeling able to share feelings with family	0.69	(0.50, 0.96)*	0.99	(0.66, 1.46)
**Total emotional support score**				
High (10–12)	0.44	(0.17, 1.12)	0.48	(0.15, 1.58)
Moderate (7–9)	0.60	(0.27, 1.36)	0.93	(0.36, 2.41)
Low (0–6) (Ref)	1.00		1.00	
***Instrumental support***				
Feeling basic needs (food/clothes) met in family	0.87	(0.54, 1.41)	0.67	(0.41, 1.12)
Feeling supported by family when sick	0.84	(0.58, 1.20)	0.68	(0.45, 1.03)
**Total instrumental support score**				
High (6)	0.63	(0.27, 1.44)	0.58	(0.22, 1.55)
Moderate (5)	0.77	(0.32, 1.87)	0.44	(0.14, 1.42)
Low (0–4) (Ref)	1.00		1.00	
***Negative interaction***				
Feeling disliked by family	1.43	(0.97, 2.10)	2.08	(1.37, 3.18)**
Feeling (emotionally) distant from family	1.37	(0.91, 2.05)	2.01	(1.27, 3.18)**
Having been physically (beaten) hurt by family member(s)	0.53	(0.23, 1.18)	0.89	(0.40, 2.00)
Feeling exploited (for housework and farming) by family	1.95	(1.30, 2.91)**	1.90	(1.21, 2.99)**
**Total negative interaction score**				
High (2–12)	3.70	(1.68, 8.18)**	5.58	(2.08, 14.96)**
Moderate (1)	0.87	(0.30, 2.51)	1.48	(0.41, 5.33)
Low (0) (Ref)	1.00		1.00	

*AOR,* adjusted odds ratio. *CI,* confidence interval. BDI, Beck Depression Inventory.

a Separate analyses were carried out for each of the ten individual items on the family support scale, adjusting as well for all variables listed in Table 2.

b After accounting for the collective set of missing values associated with the variables included in the multivariable models, data from 292 participants were analyzed in each case.

c Each individual item was assessed as a continuous variable, with responses ranging from 0 (Not at all) to 3 (All the time).

*p<0.05;

**p<0.01.

Only two family support elements were significantly associated with both mental health outcomes: *Feeling shown love and caring by family* (depression: AOR = 0.68; 95% CI = 0.46, 0.99/suicidal ideation: AOR = 0.64; 95% CI = 0.41, 0.99) and *Feeling exploited (for housework and farming) by family* (depression: AOR = 1.95; 95% CI = 1.30, 2.91/suicidal ideation: AOR = 1.90; 95% CI = 1.21, 2.99). Additionally, rates of depression were lower among those who endorsed *Feeling able to share feelings with family* (AOR = 0.69; 95% CI = 0.50, 0.96), while rates of suicidal ideation were higher among those who endorsed *Feeling disliked by family* (AOR = 2.08; 95% CI = 1.37, 3.18) and *Feeling (emotionally) distant from family* (AOR = 2.01; 95% CI = 1.27, 3.18).

### Factors Associated with Perceived Family Support

Four variables were significantly associated with perceived family support among participants: HIV symptom burden, internalized AIDS stigma, education level, and gender. Namely, those suffering from higher levels of internalized AIDS stigma (AOR = 0.35; 95% CI = 0.20, 0.60) and higher bothersome HIV symptom counts (AOR = 0.36; 95% CI = 0.21, 0.63) were less likely to report higher levels of family support. On the other side, those educated to the primary level or higher (AOR = 2.56; 95% CI = 1.42, 4.62) and men (AOR = 2.31; 95% CI = 1.22, 4.36) were more likely to report higher family support levels.

## Discussion

This study is among the first to shed light on important and distinct roles played by both positive and negative elements of perceived family support in determining the experience of serious psychological distress in the context of HIV/AIDS in a low-income Asian country. Against heavy burdens of BDI-Ia-defined depression (26%) and suicidal ideation (14%) among PLWHA in the Kathmandu Valley of Nepal, positive forms of family support – especially in the emotional realm – appeared to have a protective effect and negative family interactions an even stronger contributing effect toward both outcomes. Meanwhile, rates of depression and suicidal thoughts were elevated among those in the earlier stages of ART, those experiencing heavier burdens of HIV symptoms or stigma, those who had recently used illicit drugs, and those without employment.

Among the central findings of this study, perceived family support as a composite construct emerged as a major correlate of both depression and suicidal ideation. Indeed, those participants reporting the highest level of perceived family support were over five times less likely than those at the lowest level to register BDI-Ia-defined depression and four times less likely to endorse suicidal ideation. This result uniquely extends theory on the link between perceived family support and psychological distress to an HIV-specific population, elucidating the strong and potentially protective effect of perceived family support in terms of depression and suicidality risks. Although families generally function differently depending on cultural context, these findings point to overarching commonalities regarding the influence of perceived family support in an individual, regardless of the specific setting or the particular nature of the stressors.

Yet, based on item-wise multivariate analysis of the perceived family support scale in relation to depression and suicidal ideation, some elements of family support appear to be more important than others to the lived experience of HIV/AIDS. Namely, consistent with certain under-explored threads in research on social support and psychological well-being [20], [21], the greatest effects on the mental health outcomes were observed in the *negative* support domain, particularly with regard to suicidal ideation – an especially dangerous manifestation of psychological distress. Specifically, feelings of being exploited, rejected, and emotionally distant from family members played heavily into experiences of depression and suicidal ideation. One explanation for this may be that negative interactions are less frequently encountered and hence more saliently felt relative to positive ones. This may be particularly true in the case of family relationships. Moreover, negative interactions can potentially undermine an individual’s sense of personal control or self-worth, erode motivation to engage in positive health behaviors, and provoke adverse physiological responses [42]. Such results are in line with the domain-specific model of the link between interpersonal exchanges and mental health, in which supportive exchanges are assumed to exert a greater positive impact on positive well-being, whereas interpersonal strains are more potently manifested in negative affective states [20].

Emotional support and nurturance elements of feeling loved, cared for, and understood also emerged as important correlates in buffering against negative psychological states, whereas instrumental support factors appeared not to be as critical in determining mental health among the PLWHA surveyed. Among the positive forms of social support, emotional support is generally reported to be the most important for its clear links to health in terms of direct and buffering effects [15]. This would appear to be true across a variety of disease states, as echoed in similar findings among patients with cancer and their families [43]. Accordingly, interventions in such populations should focus on identifying and enhancing sources of emotional support even more so than more practical instrumental manifestations of assistance, particularly within family networks.

Beyond family support, four further independent variables had significant associations with both depression and suicidal ideation after adjustment: HIV symptom burden, ART treatment status, reporting any recent illicit drug use, and being unemployed. In particular, those reporting a greater number of bothersome HIV symptoms in the present study were roughly four times more likely not only to be depressed, but also to harbor suicidal thoughts. This expands significantly on prior studies of symptom-related negative mental health correlates among PLWHA [44] and points to one potential pathway for the observed association between depression and severity of HIV illness [2]. Moreover, heavier HIV symptom burdens were linked with lower levels of perceived family support in the present study, suggesting a need for interventions targeting simultaneous alleviation of symptom burden and enhancement of family support mechanisms toward improving mental health outcomes among PLWHA.

In addition to the potential psychological impacts of generalized somatic HIV-related symptoms, the results suggest that PLWHA treated with ART are more susceptible than those not on treatment to both depressive symptoms and suicidal ideation, particularly in the earlier treatment stages – perhaps when patients have not yet had time to adjust to the unique strains of treatment or to develop effective coping mechanisms that lead to reduced depression and suicidality risk over time. The observed association supports the depression-inducing potential of such treatment regimens among PLWHA, offering valuable insights into frequently overlooked psychosocial aspects of ART treatment. The undisputed benefits of ART notwithstanding, previous studies have shown that neuropsychiatric side-effects including depression are seen in up to 73% of HIV-infected patients on treatment regimens [45]. Moreover, profound changes in the lived experience and perception of illness resulting from ART introduction could potentially influence development of depression. Against the backdrop of literature detailing a knotty linkage between mental health problems and treatment outcomes, such results highlight the need for interventions to address ART side effects and for further work to identify the causes of depressive symptoms and effective ways of managing them in PLWHA on ART.

In a similar vein, both recent illicit drug use and employment status also emerged as significant correlates of both depression and suicidal ideation among the PLWHA surveyed. While such relationships with depression have previously been suggested in the general population [46], [47], evidence of the corresponding links with suicidality in PLWHA has so far been scarce. Given the serious nature of suicidal thoughts as a precursor to actual suicidal behavior, these findings demand critical attention toward adapting HIV care and treatment programs to meet identified mental health priorities. Future interventions might fruitfully explore ways to incorporate drug dependence treatment and career counseling elements, for example, toward improving overall psychological well-being among PLWHA.

Finally, depressive symptoms, though not suicidal ideation, were positively linked with both higher levels of internalized AIDS stigma and being underweight among PLWHA in this study. The correlation uncovered between depression and low BMI among PLWHA in a low-income South Asian country expands on similar findings in a study of HIV-positive men who have sex with men in the United States [48], and likely stems from the substantial weight loss that frequently comes with disease progression and/or side effects of treatment [49]. Meanwhile, the identification of stigma as a risk factor for depression is compounded by the fact that those with higher stigma scores also exhibited lower levels of perceived family support, pointing to a heightened and complexly interwoven vulnerability to depression. Notably, both mental illness and HIV/AIDS are highly stigmatized conditions in Nepal, as in many other countries – in part because of the extent to which an individual’s ailments and behavior are seen as affecting the whole family [50], [51]. Within families, forms of discrimination against PLWHA in Nepal may include restrictions on everyday activities such as movement outside the home, exposure to media, use of communal eating utensils, and access to financial resources; in some cases, family-level discrimination can even escalate to physical abuse [52], as underlined in the present study. Toward combatting such deeply ingrained elements contributing to the burden of mental illness in PLWHA, both community- and family-level anti-stigma interventions may be helpful.

### Limitations, Strengths, and Future Directions

Several study limitations should be taken into account when interpreting these results. First, although a relatively large number of participants from multiple NGO outreach networks across the Kathmandu Valley were surveyed, findings are specifically representative of PLWHA falling within the network of partnering NGOs. Second, this study relies on self-reported measures, leaving room for several sources of bias; a social desirability bias may have been introduced, for instance, in the face-to-face interview format, though a confidential and sensitive approach to survey administration was designed to limit any such tendency. Third, this study measured perceived family support rather than actual support, though research has suggested that perceived support may be even more important than objective received support or network size in its associations with health and adjustment criteria [10], [24]. Finally, moving forward, prospective studies in diverse national samples of PLWHA will help to disentangle the complex pathways that may link the interaction effects of family support elements to developing and ongoing mental health issues in this population.

Notwithstanding such limitations, the present study has clear value in testing a novel hypothesis with implications for research at the intersection of HIV/AIDS and mental health and in its use of well-validated research measures. To our knowledge, this is the first study to report the distinct effects of a set of perceived family support items – encompassing both positive (instrumental and emotional) and negative facets – on both depression and suicidal ideation among PLWHA in Nepal or elsewhere. The findings have strong implications for the development of targeted intervention and prevention efforts to address the heavy burden of psychological distress among PLWHA in such contexts. Critically, identified associations highlight the potential of programs to minimize the harmful psychological impacts of HIV-related stressors through enhancing supportive family interactions while mitigating unsupportive ones.

## Conclusions

Among PLWHA in the Kathmandu Valley of Nepal, the burden of depression and suicidal ideation is heavy and positively associated with perceived family support, among other established sociodemographic, clinical, and psychosocial correlates. Such findings underscore a need to work within a context of limited mental health resources in Nepal and similar settings by developing innovative psychosocial interventions incorporating family counseling and support elements as an integral component of HIV prevention, care, and treatment efforts. In particular, future programs should work with PLWHA to navigate the double-edged sword of family support – identifying and improving family relationships that are the source of negative exchanges while engaging positive family support structures to buffer against psychiatric comorbidities, especially in times of life stress. Given the identified correlates of perceived family support, such interventions would perhaps be most fruitful by focusing especially on female gender and low levels of education as risk factors for poor family support, and devoting special attention to those with heavy symptom burdens and high levels of internalized stigma. Coupled with this is a need for community-level anti-stigma programs to decrease the stigma and discrimination experienced both within and outside of families, thus helping to foster an environment more conducive to positively supportive interactions.

## References

[1] Sherr L, Clucas C, Harding R, Sibley E, Catalan J (2011) HIV and depression–a systematic review of interventions. Psychol Health Med 16: 493–527. 21809936 10.1080/13548506.2011.579990

[2] Rabkin JG (2008) HIV and depression: 2008 review and update. Curr HIV/AIDS Rep 5: 163–171. 18838056 10.1007/s11904-008-0025-1

[3] Shirey KG (2013) Suicide and HIV. Mental Health Practitioner’s Guide to HIV. New York: Springer New York. 405–407.

[4] Waraich P, Goldner EM, Somers JM, Hsu L (2004) Prevalence and incidence studies of mood disorders: a systematic review of the literature. Can J Psychiatry 49: 124–138. 15065747 10.1177/070674370404900208

[5] Whiteford HA, Degenhardt L, Rehm J, Baxter AJ, Ferrari AJ, et al (2013) Global burden of disease attributable to mental and substance use disorders: findings from the Global Burden of Disease Study 2010. Lancet. 10.1016/S01406736(13)616116 23993280

[6] World Health Organization (WHO) (2011) Suicide Prevention and Special Programmes. Geneva: WHO.

[7] Gonzalez JS, Batchelder AW, Psaros C, Safren SA (2011) Depression and HIV/AIDS treatment nonadherence: a review and meta-analysis. J Acquir Immune Defic Syndr 58: 181–187. 21857529 10.1097/QAI.0b013e31822d490a PMC3858003

[8] Wada N, Jacobson LP, Cohen M, French A, Phair J, et al (2013) Cause-specific life expectancies after 35 years of age for human immunodeficiency syndrome-infected and human immunodeficiency syndrome-negative individuals followed simultaneously in long-term cohort studies, 1984–2008. Am J Epidemiol 177: 116–125. 23287403 10.1093/aje/kws321 PMC3590031

[9] Ryan K, Forehand R, Solomon S, Miller C (2008) Depressive symptoms as a link between barriers to care and sexual risk behavior of HIV-infected individuals living in non-urban areas. AIDS Care 20: 331–336. 18351481 10.1080/09540120701660338

[10] Cohen S, Wills TA (1985) Stress, social support, and the buffering hypothesis. Psychol Bull 98: 310–357. 3901065

[11] Bekele T, Rourke SB, Tucker R, Greene S, Sobota M, et al (2013) Direct and indirect effects of perceived social support on health-related quality of life in persons living with HIV/AIDS. AIDS Care 25: 337–346. 22774876 10.1080/09540121.2012.701716

[12] Soykan A, Arapaslan B, Kumbasar H (2003) Suicidal behavior, satisfaction with life, and perceived social support in end-stage renal disease. Transplant Proc 35: 1290–1291. 12826139 10.1016/s0041-1345(03)00522-0

[13] McDowell TL, Serovich JM (2007) The effect of perceived and actual social support on the mental health of HIV-positive persons. AIDS Care 19: 1223–1229. 18071966 10.1080/09540120701402830 PMC2151198

[14] Li L, Lee SJ, Thammawijaya P, Jiraphongsa C, Rotheram-Borus MJ (2009) Stigma, social support, and depression among people living with HIV in Thailand. AIDS Care 21: 1007–1013. 20024757 10.1080/09540120802614358 PMC2803757

[15] Shor E, Roelfs DJ, Yogev T (2013) The strength of family ties: A meta-analysis and meta-regression of self-reported social support and mortality. Soc Networks 35: 626–638.

[16] Tezel A, Karabulutlu E, Sahin O (2011) Depression and perceived social support from family in Turkish patients with chronic renal failure treated by hemodialysis. J Res Med Sci 16: 663–673. PMC3214379 22091290

[17] Baider L, Ever-Hadani P, Goldzweig G, Wygoda MR, Peretz T (2003) Is perceived family support a relevant variable in psychological distress?. A sample of prostate and breast cancer couples. J Psychosom Res 55: 453–460. 14581100 10.1016/s0022-3999(03)00502-6

[18] Abram HS, Moore GL, Westervelt FB (1971) Suicidal behavior in chronic dialysis patients. Am J Psychiatry 127: 1199–1204. 5100612 10.1176/ajp.127.9.1199

[19] Demi A, Bakeman R, Sowell R, Moneyham L, Seals B (1998) Suicidal thoughts of women with HIV infection: effect of stressors and moderating effects of family cohesion. J Fam Psychol 12: 344–353.

[20] Okun MA, Keith VM (1998) Effects of positive and negative social exchanges with various sources on depressive symptoms in younger and older adults. J Gerontol B Psychol Sci Soc Sci 53: P4–20. 9469167 10.1093/geronb/53b.1.p4

[21] Lincoln KD (2000) Social support, negative social interactions, and psychological well-being. Soc Serv Rev 74: 231–252. 26594064 10.1086/514478 PMC4651456

[22] Ingram KM, Jones DA, Fass RJ, Neidig JL, Song YS (1999) Social support and unsupportive social interactions: their association with depression among people living with HIV. AIDS Care 11: 313–329. 10474630 10.1080/09540129947947

[23] Reinhardt JP (2001) Social support and well-being in later life: studying the negative with the positive. Appl Dev Sci 5: 66–67.

[24] Serovich JM, Kimberly JA, Mosack KE, Lewis TL (2001) The role of family and friend social support in reducing emotional distress among HIV-positive women. AIDS Care 13: 335–341. 11397335 10.1080/09540120120043982

[25] Shrestha S, Poudel KC, Poudel-Tandukar K, Kobayashi J, Pandey BD, et al (2012) Perceived Family Support and Depression among People Living with HIV/AIDS in the Kathmandu Valley, Nepal. J Int Assoc Physicians AIDS Care (Chic). 10.1177/1545109712456741 22993234

[26] WHO (2006) Mental Health System in Nepal. Kathmandu: Ministry of Health and Population Nepal.

[27] Kim HS, Sherman DK, Taylor SE (2008) Culture and social support. Am Psychol 63: 518–526. 18793039 10.1037/0003-066X

[28] Suh E, Diener E, Oishi S, Triandis HC (1998) The shifting basis of life satisfaction judgments across cultures: emotions versus norms. J Pers Soc Psychol 74: 482–493.

[29] Abe JA (2004) Self-esteem, perception of relationships, and emotional distress: a cross-cultural study. Pers Relat 11: 231–247.

[30] Amiya RM, Poudel KC, Poudel-Tandukar K, Kobayashi J, Pandey BD, et al (2011) Physicians are a key to encouraging cessation of smoking among people living with HIV/AIDS: a cross-sectional study in the Kathmandu Valley, Nepal. BMC Public Health 11: 677 10.1186/1471245811677 21878132 PMC3175193

[31] Poudel-Tandukar K, Poudel KC, Jimba M, Kobayashi J, Johnson CA, et al (2013) Serum 25-hydroxyvitamin d levels and C-reactive protein in persons with human immunodeficiency virus infection. AIDS Res Hum Retroviruses 29: 528–534. 23003113 10.1089/aid.2012.0120 PMC3581068

[32] Poudel KC, Palmer PH, Jimba M, Mizoue T, Kobayashi J, et al (2013) Coinfection of hepatitis C virus among HIV-positive people in the Kathmandu Valley, Nepal. J Int Assoc Provid AIDS Care. 10.1177/2325957413500989 24056798

[33] United Nations Development Programme (UNDP) (2013) Human Development Report 2013. The Rise of the South: Human progress in a diverse world. New York: UNDP.

[34] National Centre for AIDS and STD Control (NCASC) (2012) National estimates of HIV infections in Nepal 2012. Kathmandu: NCASC.

[35] NCASC (2007) National estimates of HIV infections, Nepal. Kathmandu: NCASC.

[36] Central Bureau of Statistics (CBS) (2011) Population Census 2011, Preliminary Results. Kathmandu: CBS.

[37] Kohrt BA, Kunz RD, Koirala NR, Sharma VD, Nepal MK (2002) Validation of a Nepali version of the Beck Depression Inventory. Nep J Psychiatry 2: 123–130.

[38] Kohrt BA, Speckman RA, Kunz RD, Baldwin JL, Upadhaya N, et al (2009) Culture in psychiatric epidemiology: using ethnography and multiple mediator models to assess the relationship of caste with depression and anxiety in Nepal. Ann Hum Biol 36: 261–280. 19381985 10.1080/03014460902839194 PMC3907946

[39] WHO Expert Consultation (2004) Appropriate body-mass index for Asian populations and its implications for policy and intervention strategies. Lancet 363: 157–163. 14726171 10.1016/S0140-6736(03)15268-3

[40] Justice AC, Holmes W, Gifford AL, Rabeneck L, Zackin R, et al (2001) Development and validation of a self-completed HIV symptom index. J Clin Epidemiol 54 Suppl 1: S77–90. 11750213 10.1016/s0895-4356(01)00449-8

[41] Simbayi LC, Kalichman S, Strebel A, Cloete A, Henda N, et al (2007) Internalized stigma, discrimination, and depression among men and women living with HIV/AIDS in Cape Town, South Africa. Soc Sci Med 64: 1823–1831. 17337318 10.1016/j.socscimed.2007.01.006 PMC4271649

[42] Cohen S (1988) Psychosocial models of the role of social support in the etiology of physical disease. Health Psychol 7: 269–297. 3289916 10.1037//0278-6133.7.3.269

[43] Kaushik S, Arun P (2010) Psychiatric morbidity and psychotherapeutic interventions in cancer survivors. J Mental Health Hum Behav 15: 77–87.

[44] Gaynes BN, Pence BW, Atashili J, O’Donnell J, Kats D, et al (2012) Prevalence and predictors of major depression in HIV-infected patients on antiretroviral therapy in Bamenda, a semi-urban center in Cameroon. PLoS One 7: e41699. 22860006 10.1371/journal.pone.0041699 PMC3409230

[45] Kaestner F, Anneken K, Mostert C, Reichelt D, Rothermundt M, et al (2012) Depression associated with antiretroviral drug therapy in HIV: case report and overview. Int J STD AIDS 23: e14–19. 10.1258/ijsa.2009.009451 22807551

[46] Center for Substance Abuse Treatment (2008) Substance Abuse and Suicide Prevention: Evidence and Implications - A White Paper. Rockville: Substance Abuse and Mental Health Services Administration.

[47] Milner A, Page A, LaMontagne AD (2013) Long-term unemployment and suicide: a systematic review and meta-analysis. PLoS One 8: e51333. 23341881 10.1371/journal.pone.0051333 PMC3547020

[48] Blashill AJ, O’Cleirigh C, Mayer KH, Goshe BM, Safren SA (2012) Body mass index, depression and sexual transmission risk behaviors among HIV-positive MSM. AIDS Behav 16: 2251–2256. 21983696 10.1007/s10461-011-0056-2 PMC3623671

[49] Siddiqui J, Phillips AL, Freedland ES, Sklar AR, Darkow T, et al (2009) Prevalence and cost of HIV-associated weight loss in a managed care population. Curr Med Res Opin 25: 1307–1317. 19364303 10.1185/03007990902902119

[50] Kohrt BA, Hruschka DJ (2010) Nepali concepts of psychological trauma: the role of idioms of distress, ethnopsychology and ethnophysiology in alleviating suffering and preventing stigma. Cult Med Psychiatry 34: 322–352. 20309724 10.1007/s11013-010-9170-2 PMC3819627

[51] Nepal VP, Ross MW (2010) Issues related to HIV stigma in Nepal. Int J Sex Health 22: 20–31.

[52] Neupane D, Khanal V, Sharma S, Aro AR (2012) Perceived discrimination among people living with HIV in Nepal. J Nepal Health Res Counc 10: 136–140. 23034376

